# Relation between presence of extended-spectrum β-lactamase-producing *Enterobacteriaceae* in systematic rectal swabs and respiratory tract specimens in ICU patients

**DOI:** 10.1186/s13613-017-0237-x

**Published:** 2017-02-02

**Authors:** Hélène Carbonne, Matthieu Le Dorze, Anne-Sophie Bourrel, Hélène Poupet, Claire Poyart, Emmanuelle Cambau, Jean-Paul Mira, Julien Charpentier, Rishma Amarsy

**Affiliations:** 1 Service de Réanimation Chirurgicale Polyvalente, Département d’Anesthésie Réanimation SMUR, Hôpital Lariboisière, AP-HP 2, Rue Ambroise Paré, 75475 Paris Cedex 10, France; 2 Laboratoire de Microbiologie, Hôpital Cochin, AP-HP, 27 rue du Faubourg Saint-Jacques, 75014 Paris, France; 3 Laboratoire de Bactériologie-Virologie, Hôpital Lariboisière, AP-HP, 2 Rue Ambroise Paré, 75475 Paris Cedex 10, France; 4 Service de Réanimation Médicale, Hôpital Cochin, AP-HP, 27 rue du Faubourg Saint-Jacques, 75014 Paris, France; 5 Equipe Opérationnelle d’Hygiène, Hôpital Lariboisière, AP-HP, 2 Rue Ambroise Paré, 75475 Paris Cedex 10, France

**Keywords:** *Enterobacteriaceae*, Extended-spectrum β-lactamase, Multidrug resistance, Digestive colonization, Respiratory sample, Intensive care unit

## Abstract

**Background:**

The choice of empirical antimicrobial therapy for pneumonia in intensive care unit (ICU) is a challenge, since pneumonia is often related to multidrug-resistant pathogens, particularly extended-spectrum β-lactamase-producing *Enterobacteriaceae* (ESBL-E). To prevent the overuse of broad-spectrum antimicrobial therapy, the main objective of this study was to test the performance of digestive colonization surveillance as a predictor of ESBL-E presence or absence in respiratory samples performed in ICU and to evaluate the impact of time sampling (≤5 days or >5 days) on such prediction. Design: Multicentric retrospective observational study, including every patient with a respiratory tract specimen positive culture and a previous rectal ESBL-E screening performed within 7 days before the respiratory sample, between January 2012 and December 2014. Results were analyzed in two groups: respiratory samples obtained during the first 5 days of ICU stay (early group) and respiratory samples obtained after 5 days (late group). Interventions: none.

**Results:**

Among 2498 respiratory tract samples analyzed corresponding to 1503 patients, 1557 (62.3%) were performed early (≤5 days) and 941 (37.7%) later (>5 days). Positivity rates for ESBL-E were 15.0 and 36.8% for rectal swabs in the early and late groups, respectively. Sensitivity, specificity, positive (PPV) and negative (NPV) predictive values and likelihood ratios were calculated for ESBL-E digestive colonization as a predictor of ESBL-E presence in respiratory samples. PPVs of ESBL-E digestive colonization were 14.5% (95% CI [12.8; 16.3]) and 34.4% (95% CI [31.4; 37.4]), for the early and late groups, respectively, whereas NPVs were 99.2% (95% CI [98.7; 99.6]) and 93.4% (95% CI [91.9; 95.0]), respectively.

**Conclusions:**

Systematic surveillance of ESBL-E digestive colonization may be useful to limit the use of carbapenems when pneumonia is suspected in ICU. When rectal swabs are negative, the risk of having ESBL-E in respiratory samples is very low even after 5 days of ICU stay.

**Electronic supplementary material:**

The online version of this article (doi:10.1186/s13613-017-0237-x) contains supplementary material, which is available to authorized users.

## Background

Community-acquired, hospital-acquired and ventilator-associated pneumonia (VAP) are the most common infections in intensive care units (ICU). They are associated with high morbidity and mortality rates [1, 2], particularly if the administration of appropriate antimicrobial therapy is delayed [3–6]. The choice of empirical antimicrobial therapy is a challenge since it can only be validated a posteriori when sample cultures and antibiotic susceptibility testing are known [7]. Because of frequent long hospital stays, complex underlying pathologies and previous antimicrobial exposure, pneumonia is often related to multidrug-resistant (MDR) pathogens, particularly extended-spectrum β-lactamase-producing *Enterobacteriaceae* (ESBL-E) [8, 9]. Incidence of ESBL-E is increasing, 15% of patients admitted in ICU have an ESBL-E digestive colonization in a French study conducted between 2010 and 2011 [10]. The use of local epidemiological data and individual patient risk factors leads to frequent empirical prescription of broad-spectrum antimicrobial therapy, including carbapenems [11–13], leading to the emergence of MDR pathogens [14], especially carbapenemase-producing *Enterobacteriaceae* [15].

To prevent the overuse of such broad-spectrum antimicrobial therapy, rapid susceptibility testing [16–18] and colonization monitoring [19–21] have been developed, aiming to administer adequate treatment as early as possible. We have previously shown that microbiological examination of upper airways samples at ICU admission predicts the microorganisms involved in VAP occurring in the early course of a patient’s ICU stay with a high specificity and likelihood ratio [22].

The objectives of this study were: (1) to test the performance of digestive colonization surveillance as a predictor of ESBL-E presence or absence in respiratory samples performed in ICU; (2) to evaluate the impact of time sampling (≤5 days or >5 days) on such prediction; (3) to verify the impact of a medical versus surgical population on the results. We hypothesized that a systematic detection of ESBL-E digestive colonization may help to limit the use of carbapenems.

## Methods

### Study design and inclusion criteria

From January 2012 to December 2014, a multicentric retrospective observational study was performed in two teaching hospitals’ adult ICUs in Paris: the 21-bed surgical ICU at Lariboisière Hospital and the 24-bed medical ICU at Cochin Hospital. In each center, an infection prevention and control team ensured that appropriate infection prevention and management strategies were implemented, evaluated for effectiveness and modified it, in agreement with the national surveillance network coordinated by the RAISIN (Réseau d’Alerte d’Investigation et de Surveillance des Infections Nosocomiales). Since rectal swabs and respiratory samples were part of our daily practice and no intervention was tested, the Ethics Committee of French Society of Intensive Care (*Société de Réanimation de Langue Française*, CE SRLF 15-30) approved the protocol and waived the requirement of written informed consent. Furthermore, a declaration to the *Commission Nationale de l’Informatique et des Libertés* (CNIL) was done (declaration number: 1880024).

During the study period, patients having a respiratory specimen with a positive culture of any bacteria, including ESBL-E, were enrolled (see below for microbiological criteria). Respiratory samples were performed only in case of VAP suspicion in the surgical ICU, whereas systematic endotracheal aspirate surveillance cultures [19] were performed in the medical ICU. Patients with no previous rectal swab available within 7 days before the respiratory sampling were excluded. When duplicate respiratory samples were obtained within 48 h and were positive with the same pathogen, only one of them was included. Early respiratory samples corresponded to samples obtained during the first 5 days of ICU stay, defining the “early group.” Late respiratory samples corresponded to samples performed after 5 days of ICU stay, defining the “late group.” Clinical characteristics were collected to describe the population: age, sex ratio, simplified acute physiology score II (SAPS II), ICU mortality rate, length of stay in ICU, duration of mechanical ventilation and main admission diagnosis.

#### Microbiology

The microbiological methods were similar in the two centers (same swab, same medium, same inoculum device and same antibiotic susceptibility testing).

##### Rectal ESBL-E screening

Rectal ESBL-E screening was routinely performed within the first 24 h after ICU admission and weekly thereafter. Rectal swabs were performed by nurses using ESwab^®^ (COPAN Diagnostics, Italy). Transport medium was then inoculated using PREVI^®^ Isola standardized inoculation system (BioMérieux, Marcy-L’Etoile, France) on selective chromogenic ChromID^®^ ESBL agar plates (BioMérieux, Marcy-L’Etoile, France). Growing colonies were identified after 24 h of 37 °C aerobic conditions incubation using mass spectrometry with MALDI™ Biotyper system (Bruker Daltonics, Germany). Antimicrobial susceptibility was tested by disk diffusion method with Mueller–Hinton agar plates (MH agar plates, BioMérieux, Marcy-L’Etoile, France) according to the EUCAST (European Committee on Antimicrobial Susceptibility Testing) and CA-SFM (Antibiogram Committee of the French Society of Microbiology) guidelines [23]. ESBL-E digestive colonization was defined by one or more ESBL-E strain isolated from a rectal swab.

##### Respiratory samples

Respiratory samples were endotracheal aspirates (Unomedical, ConvaTec, Deeside, United Kingdom), sputum samples obtained by expectoration after oral care with the assistance of a physiotherapist when necessary, protected distal sampling (Combicath, Plastimed, Le Plessis Bouchard, France) using a fiberoptic bronchoscope, and bronchoalveolar lavages (BAL) during bronchoscopy by slowly injecting and retrieving from the lung area of interest 100 mL of isotonic saline. Samples were isolated on agar plates using routine methods according to the French Society of Microbiology guidelines [23]. Microbiological identification and antimicrobial susceptibility testing were obtained as described above. Respiratory sample was defined as positive when at least 10^3^ colony-forming units (CFU)/mL were observed in protected distal sampling, 10^4^ CFU/mL in BAL, 10^6^ (CFU)/mL in endotracheal aspirates and 10^7^ CFU/mL in sputum cultures. Culture results with microbiological identification and resistance patterns were reported to the treating physicians within 2 days after sampling. Focus was made on presence or absence of ESBL-E in the respiratory sample and in the previous rectal swab, regardless of the *Enterobacteriaceae* species.

### Statistical analysis

Quantitative variables were described using median (interquartile range) or mean (standard deviation) and categorical variables using number (percentage). Proportions were compared using the Chi-square test. Continuous variables were compared by the Student *t* test. Nonparametric variables were compared using the Mann–Whitney test. Sensitivity, specificity, positive predictive value (PPV), negative predictive value (NPV) and likelihood ratios (LR) were obtained by standard statistical methods. Prism Software^®^ (GraphPad Software^®^, La Jolla, USA) was used for the statistical analysis.

## Results

### Population characteristics

Demographic data of all patients (*n* = 1503), medical ICU patients (*n* = 1147) and surgical ICU patients (*n* = 356) are described in Table 1. The two populations clearly differed, the medical ICU patients being older, more severe at admission, with a higher mortality rate and a shorter length of stay.

**Table 1:** Demographic data

Variable	All patients (*n* = 1503)	Surgical ICU patients (*n* = 356)	Medical ICU patients (*n* = 1147)	*p*
Age, *year*	63 (±17)	59 (±17)	64 (±17)	<0.0001
Gender, male *n* (%)	983 (65.4)	230 (64.6)	753 (65.6)	0.7179
SAPS II, points	55 (±20.8)	42.2 (±13.9)	59.5 (±21)	<0.0001
ICU mortality, *n* (%)	328 (21.8)	57 (16.0)	271 (23.6)	0.0021
Days of ICU^a^ hospitalization, *n*	8 (3–19)	15 (7–26)	7 (3–15)	<0.0001
Patients under MV, *n* (%)	1264 (84.1)	326 (91.6)	938 (81.8)	<0.0001
Days of MV, *n*	6 (2–13)	10 (4–19)	5 (2–10)	<0.0001
Main admission diagnosis, *n* (%)				
Respiratory distress	501 (33.3)	74 (20.8)	427 (37.2)	<0.0001
Neurological failure	407 (27.1)	175 (49.2)	232 (20.2)	<0.0001
Cardiac arrest	196 (13.0)	1 (0.3)	195 (17.0)	<0.0001
Sepsis or septic shock	122 (8.1)	24 (6.7)	98 (8.6)	0.2767
Other	277 (18.5)	82 (23.0)	195 (17.0)	0.0103

Data are expressed as absolute values (percentage), mean (standard deviation) or median (interquartile range)

*ICU* Intensive care unit, *SAPS II* Simplified Acute Physiology Score II, *MV* Mechanical ventilation

*p* statistical difference between patients from medical and surgical ICU

#### Respiratory samples

**Fig. 1:**
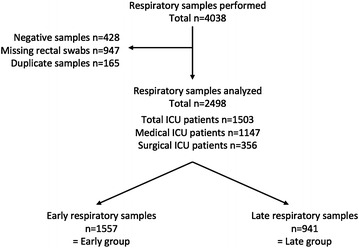
Flowchart. *ICU* intensive care unit

A total of 4038 respiratory samples were performed among which 3610 (89.4%) were culture-positive. Among them, 1112 respiratory samples were excluded: 947 samples with missing rectal swabs, and 165 duplicate samples for which only one sample was included. Finally, 2498 respiratory samples were obtained on 1503 patients. These samples were divided in 1557 (62.3%) early samples (≤5 days) and 941 (37.7%) late samples (>5 days) (Fig. 1). A total of 2073 and 425 respiratory samples were, respectively, collected in medical ICU (Cochin Hospital) and in surgical ICU (Lariboisière Hospital). Early respiratory samples (≤5 days, *n* = 1557) were performed during mechanical ventilation in 79.6% of cases after a median delay of 1 (0–2) day of ICU stay. Late respiratory samples (>5 days, *n* = 941) were performed during mechanical ventilation in 90.4% of patients after a median delay of 13 (8–23) days of ICU stay. Respiratory samples techniques were endotracheal aspirates (*n* = 2122, 85.0%), sputum cultures (*n* = 240, 9.6%), BAL (*n* = 77, 3.1%) and distal protected aspirates (*n* = 59, 2.3%).

### Microbiological epidemiology

ESBL-E prevalence in rectal swabs and respiratory samples are described in Table 2.

**Table 2:** Extended-spectrum β-lactamase-producing *Enterobacteriaceae* prevalence in rectal swabs and respiratory samples in the early and late groups

Early group (*n* = 1557)	Respiratory sample ESBL-E (+) 45/1557 (2.9%)	Respiratory sample ESBL-E (−) 1512/1557 (97.1%)
*Rectal swab ESBL*-*E* (+) 234/1557 (15.0%)	34	200
*Rectal swab ESBL*-*E* (−) 1323/1557 (85.0%)	11	1312

Late group (*n* = 941)	Respiratory sample ESBL-E (+) 158/941 (16.8%)	Respiratory sample ESBL-E (−) 783/941 (83.2%)
*Rectal swab ESBL*-*E* (+) 346/941 (36.8%)	119	227
*Rectal swab ESBL*-*E* (−) 595/941 (63.2%)	39	556

Prevalence is expressed as absolute value (percentage). ESBL: extended-spectrum β-lactamase-producing *Enterobacteriaceae*. The early group is defined by respiratory samples collected within the first 5 days after intensive care unit (ICU) admission, and the late group is defined by respiratory samples collected after 5 days of ICU hospitalization

**Fig. 2:**
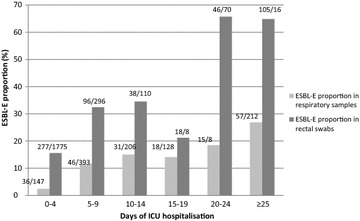
Proportion of extended-spectrum β-lactamase-producing *Enterobacteriaceae* among all rectal swabs and positive respiratory samples performed during 5 days periods. *ESBL*-*E* extended-spectrum β-lactamase-producing *Enterobacteriaceae*; *ICU* intensive care unit

In the early group, 15.0% of rectal swabs were positive for ESBL-E, and only 14.5% of them corresponded with a respiratory sample positive for ESBL-E. In the late group, 36.8% of rectal swabs were positive for ESBL-E and 34.4% of them corresponded with a respiratory sample positive for ESBL-E. The prevalence of ESBL-E in rectal swabs and in respiratory samples was not statistically different between the two study centers (Additional file 1: Table S1). Concerning the rectal swabs positive for ESBL-E, the main species identified were *Escherichia coli* (40.0%), *Klebsiella pneumoniae* (24.0%) and *Enterobacter cloacae* (18.1%). With the demographic data collected, we did not highlight any risk factors of having a rectal swab positive for ESBL-E in both the early and late group (data not shown). Concerning the respiratory samples, *Enterobacteriaceae* represented the main species identified both in early and late group. The prevalence of ESBL-E in rectal swabs and in respiratory samples increased significantly in the late group compared to the early group (*p* < 0.0001). Figure 2 depicts the evolution of proportion of ESBL-E positive rectal swabs and respiratory samples with ICU length of stay. The proportion of ESBL-E positive respiratory samples increased versus time, when proportion of positive rectal swabs seemed stable until day 20, between 15 and 34%.

## Performance characteristics of ESBL-E digestive colonization as a predictor of ESBL-E presence or absence in respiratory samples

Table 3 summarized the sensitivity, specificity, predictive values and likelihood ratios of ESBL-E digestive colonization as a predictor of ESBL-E presence or absence in respiratory samples performed early and late after ICU admission. For the early group, PPV for ESBL-E digestive colonization was 14.5% (95% CI [12.8; 16.3]), and NPV was 99.2% (95% CI [98.7; 99.6]). For the late group, PPV for ESBL-E digestive colonization was 34.4% (95% CI [31.4; 37.4]), and NPV was 93.4% (95% CI [91.9; 95.0]). These results were not statistically different between the two study centers (Additional file 1: Table S2). With the data collected, we did not highlight any risk factors of having a respiratory sample positive for ESBL-E when the rectal swab was positive for ESBL-E (data not shown).

**Table 3:** Sensitivity, specificity, positive predictive value, negative predictive value and likelihood ratios of digestive colonization for extended-spectrum β-lactamase-producing *Enterobacteriaceae* in respiratory sample

Variable	Early group (≤5 days) (*n* = 1557)	Late group (>5 days) (*n* = 941)
Sensitivity (%) [95% CI]	75.6% [73.4–77.7]	75.3% [72.6–78.1]
Specificity (%) [95% CI]	86.8% [85.1–88.5]	71.0% [68.1–73.9]
Positive predictive value (%) [95% CI]	14.5% [12.8–16.3]	34.4% [31.4–37.4]
Negative predictive value (%) [95% CI]	99.2% [98.7–99.6]	93.4% [91.9–95.0]
Positive LR [95% CI]	5.71 [4.63–7.05]	2.60 [2.26–2.99]
Negative LR [95% CI]	0.28 [0.17–0.47]	0.35 [0.26–0.46]

*LR* likelihood ratio. Sensitivity, specificity, positive predictive value, negative predictive values are expressed as percentage [95% CI]. Likelihood ratios are expressed as absolute value [95% CI]

## Discussion

The appropriateness of empirical antimicrobial therapy for pneumonia is a critical issue in ICU. Current guidelines suggest to use local epidemiological data and individual patient’s risk factors to guide probabilistic antimicrobial therapy [11]. This may lead to the overuse of broad-spectrum antimicrobial therapy, particularly carbapenems. In the present study, we hypothesized that a systematic detection of ESBL-E digestive colonization may help to limit the use of carbapenems despite the high incidence of MDR pathogens risk factors in ICU. It is presumed that bacterial flora changes during ICU stay with colonization of the upper airway by digestive flora [24]. The performance of rectal swab to predict EBSL-E presence or absence in respiratory samples was then investigated in early (≤5 days) and late (>5 days) period after ICU admission. Moreover, this approach could be pragmatic taking into account the evolution of inflammatory patterns along time with an initial intense inflammatory response that may result in organ dysfunction and early death, followed by a later phase characterized by a post-aggressive immunosuppression [25]. Medical and surgical ICU patients were investigated, insuring good external validity. The main results were: (1) Medical and surgical patients had similar prevalence of EBSL-E in rectal swab and in respiratory samples, despite very different clinical characteristics; (2) the early and late groups showed very different prevalence of EBSL-E in rectal swabs and respiratory samples; (3) when rectal swabs were negative, the risk of having ESBL-E in respiratory samples was very low for both early and late groups.

The prevalence of positive rectal swab was similar to the previously reported data, reaching 15.0% in rectal swabs performed before day 5 [10] and 36.8% in rectal swabs performed after day 5. This prevalence was higher than the one found by Bruyère et al. (6.8%) in a study conducted in France between 2006 and 2013 [21].

Early after ICU admission (≤5 days), when the rectal swab was negative for ESBL-E, respiratory samples were also negative for ESBL-E in 99.2% of cases. This may help reduce the prescription of carbapenems when pneumonia is suspected. When the rectal swab was positive for ESBL-E, only 14.5% of respiratory samples were positive for ESBL-E. As the patient may develop a pro-inflammatory response secondarily to pneumonia at the early phase of sepsis, the risk would be too high not to use carbapenems when the rectal swab is positive for ESBL-E and the clinical condition is severe. These patients might present a particular condition such as long-term hospitalization or iterative use of antimicrobial drugs selecting ESBL-E in their digestive flora. It would be interesting to identify risk factors of having a respiratory sample positive for ESBL-E when rectal swab is positive for ESBL-E in a patient cohort with pneumonia.

Late rectal swabs, performed after 5 days of ICU admission, showed a higher incidence of EBSL-E (36.8%) than early rectal swabs (15.0%). Among these samples, interestingly the NPV remained very good (93.4%) despite the presence of several MDR pathogens risk factors since these patients are hospitalized in ICU for a median of 13 days with a likely previous antimicrobial therapy. This may also help reduce the prescription of carbapenems when pneumonia is suspected. When the rectal swab was positive for ESBL-E, 34.4% of respiratory samples were also positive for ESBL-E, a relatively high incidence. Since these samples were related to the late phase of ICU stay, a sensible solution would be to wait for the microbiological results before antimicrobial therapy initiation when the clinical condition is not life threatening. As a consequence, overuse of carbapenems may be avoided in the late phase, with minimal individual risk and better control of MDR pathogens selection risk.

**Fig. 3:**
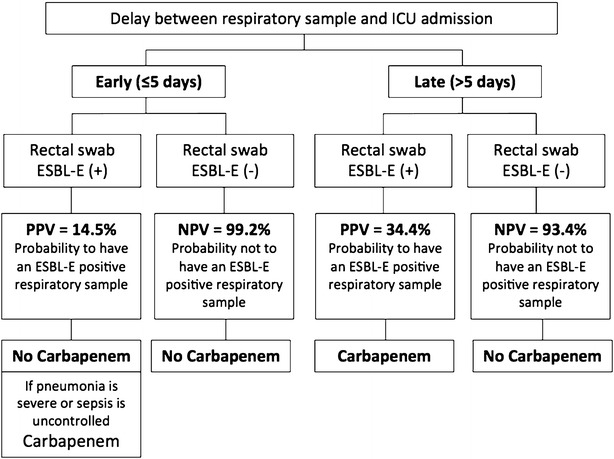
Suggestion of decision tree for empirical antimicrobial therapy in patients with respiratory tract specimen positive culture and suspicion of pneumonia. Suggestion of decision tree to limit the use of carbapenems in the setting of empirical antimicrobial therapy in patients with respiratory tract specimen positive culture and suspicion of pneumonia. By “No Carbapenem,” the authors mean another empirical antimicrobial therapy based on local epidemiological data and the American Thoracic Society guidelines [11]. In the situation of an early positive respiratory tract specimen culture with previous ESBL-E positive rectal swab, the choice of empirical antimicrobial therapy should take into account patient’s severity and clinical condition. *ICU* intensive care unit, *ESBL*-*E* extended-spectrum β-lactamase-producing *Enterobacteriaceae*; *PPV* positive predictive value, *NPV* negative predictive value

Relying on our results and on the American thoracic society guidelines [11], we suggest a decision tree for empirical antimicrobial therapy in patients with respiratory tract specimen positive culture and suspicion of pneumonia (Fig. 3).

One can argue that the very high NPV found in early (99.2% [98.7–99.6]) and late (93.4% [91.9–95.0]) groups were due to the low prevalence of ESBL-E in respiratory samples. However, in this study, the overall prevalence of ESBL-E in early and late respiratory samples was 8.1% (203/2498), which was higher than the prevalence described in the French ICU nosocomial infection surveillance network in 2013 (6.4%) and 2014 (4.9%) [26, 27]. Consequently, our results can be generalized to ICUs with roughly the same ESBL-E prevalence in respiratory tract specimens’ cultures.

The link between rectal swabs and respiratory samples positive for EBSL-E is based on a debatable hypothesis, suggesting a contamination from digestive flora to the respiratory tract [24]. The evolution of ESBL-E positive samples proportion versus duration of ICU stay revealed surprising results. From day 0 to day 20, the incidence of ESBL-E positive rectal swabs remained less than 35%, whereas the incidence of ESBL-E positive respiratory samples was increasing along time in a linear trend. This supports the idea of an increased respiratory colonization by EBSL-E from digestive flora. One can then hypothesize that the delay between the first ESBL-E positive rectal swab and the respiratory sample might guide the decision to use carbapenems. If the first ESBL-E positive rectal swab is early during the hospitalization, there might be a high risk to observe an EBSL-E positive respiratory sample. On the contrary, if rectal swabs were negative for several days with a recent ESBL-E positive rectal swab, there might be a low risk of ESBL-E in the respiratory sample.

However, there are some limitations to our study. As high as 27% of the culture-positive samples have been excluded due to the absence of rectal swab. Moreover, the weight of patients who had multiple respiratory sampling may have influenced the results. The evidence of a clinical benefit of ESBL-E digestive colonization surveillance to predict the presence or the absence of ESBL-E in respiratory samples needs to be confirmed in a clinical study involving pneumonia and not only respiratory samples. As we chose to focus on respiratory samples and not episodes of pneumonia, we did not study antimicrobial therapy regimens. We chose to focus on ESBL-E presence or absence in respiratory samples and in previous rectal swabs, regardless of the *Enterobacteriaceae* species. Indeed, the association of ESBL-E presence or absence in rectal swab and respiratory sample is relevant for the choice of empirical antimicrobial therapy, whatever the *Enterobacteriaceae* species. In addition, ESBL-E are transmitted through plasmids from an *Enterobacteriaceae* strain to another, making the concordance interpretation between respiratory and rectal ESBL-E difficult.

## Conclusions

Systematic surveillance of ESBL-E digestive colonization may be useful to limit the use of carbapenems when pneumonia is suspected, particularly in the late phase of ICU stay. When the rectal swab is negative for ESBL-E, whatever the length of stay in ICU, carbapenems may not be used. The evidence of clinical benefit of ESBL-E digestive colonization surveillance to predict the presence or the absence of ESBL-E in respiratory samples needs to be confirmed in a large prospective clinical study.

## Additional file

**Additional file 1: Table S1.** Comparison of the two study centers ESBL-E prevalence in rectal swabs and respiratory samples in the early and late groups. **Table S2.** Comparison of sensitivity, specificity, positive predictive value, negative predictive value and likelihood ratios of digestive colonization for ESBL-E in respiratory sample in the two centers.

## Abbreviations

BAL: bronchoalveolar lavage
CFU: colony-forming units
CI: confidence interval
ESBL-E: extended-spectrum β-lactamase-producing *Enterobacteriaceae*
ICU: intensive care unit
LR: likelihood ratio
MDR: multidrug-resistant pathogens
NPV: negative predictive value
PPV: positive predictive value
SAPS II: simplified acute physiology score II
VAP: ventilator-associated pneumonia
