# Supplementary material for: Relation between presence of extended-spectrum β-lactamase-producing Enterobacteriaceae in systematic rectal swabs and respiratory tract specimens in ICU patients
Source: Ann Intensive Care. 2017 Feb 2;7:13. doi: 10.1186/s13613-017-0237-x (PMC5289933; doi:10.1186/s13613-017-0237-x)
Supplement: Supplementary file 1 — Additional file 1: Table S1. Comparison of the two study centers ESBL-E prevalence in rectal swabs and respiratory samples in the early and late groups. Table S2. Comparison of sensitivity, specificity, positive predictive value, negative predictive value and likelihood ratios of digestive colonization for ESBL-E in respiratory sample in the two centers. [file 13613_2017_237_MOESM1_ESM.docx]

**Supplemental digital content table 1.** Comparison of the two study centers ESBL-E prevalence in rectal swabs and respiratory samples in the early and late groups

|  | **Surgical ICU (n=425)** | **Medical ICU (n=2073)** | ***P*** |
| --- | --- | --- | --- |
| **Early group** | | |  |
| Rectal swab ESBL-E (+) | 32/231 (13.9%) | 202/1326 (15.2%) | *P*=0.5878 |
| Respiratory sample ESBL-E (+) | 3/231 (1.3%) | 42/1326 (3.2%) | *P*=0.1177 |
| **Late group** | | |  |
| Rectal swab ESBL-E (+) | 66/194 (34.0%) | 280/747 (37.5%) | *P*=0.3728 |
| Respiratory sample ESBL-E (+) | 26/194 (13.4%) | 132/747 (17.7%) | *P*=0.5878 |
| *Proportions were compared using the chi-square test. Prevalence is expressed as absolute value (percentage). ESBL-E: extended-spectrum β-lactamase producing Enterobacteriaceae* | | | |

**Supplemental digital content table 2.** Comparison of sensitivity, specificity, positive predictive value, negative predictive value and likelihood ratios of digestive colonization for ESBL-E in respiratory sample in the two centers.

|  | | | | | | |
| --- | --- | --- | --- | --- | --- | --- |
|  | **Early group (≤5 days)** | | | **Late group (>5 days)** | | |
|  | Surgical ICU (n=231) | Medical ICU (n=1326) | *P* | Surgical ICU (n=194) | Medical ICU (n=747) | *P* |
| Sensitivity (%) [95% CI] | 66.7% [60.6-72.7] | 76.2% [73.9-78.5] | *P*=0.7108 | 80.7% [75.2-86.3] | 74.2% [71.1-77.4] | *P*=0.4805 |
| Specificity (%) [95% CI] | 86.8% [82.5-91.2] | 86.8% [84.9-88.6] | *P*=0.9731 | 73.2% [67.0-79.5] | 70.4% [67.1-73.7] | *P*=0.4772 |
| PPV (%) [95% CI] | 6.3% [3.1-9.4] | 15.9% [13.9-17.8] | *P*=0.1526 | 31.8% [25.3-38.4] | 35.0% [31.6-38.4] | *P*=0.6245 |
| NPV (%) [95% CI] | 99.5% [98.6-100.0] | 99.1% [98.6-99.6] | *P*=0.5793 | 96.1% [93.4-98.8] | 92.7% [90.9-94.6] | *P*=0.1718 |
| Positive LR [95% CI] | 5.07 [2.13-12.06] | 5.76 [4.62-7.17] |  | 3.02 [2.21-4.12] | 2.51 [2.14-2.94] |  |
| Negative LR [95% CI] | 0.38 [0.08-1.90] | 0.27 [0.16-0.47] |  | 0.27 [0.12-0.58] | 0.37 [0.27-0.50] |  |
| *LR: likelihood ratio. Sensitivity, specificity, positive predictive value, negative predictive value are expressed as percentage [95% CI]. Likelihood ratios are expressed as absolute value [95% CI]. Proportions were compared using the chi-square test.* | | | | | | |
